# Impact of platelet transfusions on plasma proteomes in controlled endotoxemia and in hemato‐oncological patients

**DOI:** 10.1111/trf.18397

**Published:** 2025-09-03

**Authors:** Eva R. Smit, Isabella Viegen, Pieter F. van der Meer, Stefan F. van Wonderen, Floor L. F. van Baarle, Paula F. Ypma, Jean‐Louis H. Kerkhoffs, Jan Voorberg, Alexander P. J. Vlaar, Anna L. Peters, Maartje van den Biggelaar, Diana Muñoz Sandoval

**Affiliations:** ^1^ Department of Molecular Hematology Sanquin Research Amsterdam the Netherlands; ^2^ Department of Intensive Care Amsterdam UMC location University of Amsterdam Amsterdam the Netherlands; ^3^ Department of Hematology Haga Teaching Hospital The Hague the Netherlands; ^4^ Department of Product and Process Development Sanquin Blood Bank Amsterdam the Netherlands; ^5^ Laboratory of Experimental Intensive Care and Anesthesiology Amsterdam UMC location University of Amsterdam Amsterdam the Netherlands; ^6^ Unit Transfusion Medicine Sanquin Blood Bank Amsterdam The Netherlands; ^7^ Department of Experimental Vascular Medicine Amsterdam UMC location University of Amsterdam Amsterdam the Netherlands

**Keywords:** controlled endotoxemia, hemato‐oncology, plasma, platelet, proteomics, transfusion

## Abstract

**Background:**

Inflammation is a hallmark of patients that receive platelet transfusions, including critically ill and hemato‐oncological patients. Platelet transfusions have been suggested to exacerbate inflammatory conditions, resulting in transfusion‐related complications. Here, we used plasma proteomics to study the impact of platelet transfusions under inflammatory conditions.

**Methods:**

Plasma proteomics was performed in samples from two studies: (1) 252 samples from healthy volunteers from 36 cases divided into groups (*n* = 6) with and without controlled endotoxemia, each with different platelet products, and (2) 54 samples from 27 transfusion events in hemato‐oncological patients (*n* = 14). Both studies had samples pre‐ and post‐transfusions.

**Results:**

Controlled endotoxemia elicited a shared inflammatory response across all healthy volunteers. This response was characterized by increased abundance of two kinetically distinct protein clusters, an initial cluster associated with degranulation and inflammation (i.e., S100A9 and MMP9) followed by a cluster of acute‐phase proteins (i.e., SAA and CRP). Autologous platelet transfusions in healthy individuals did not induce transfusion‐specific changes in plasma protein levels, nor did they exacerbate the effect of controlled endotoxemia. In hemato‐oncological patients, we found a transfusion‐specific response restricted to alterations in platelet basic protein (PPBP) levels. No additional changes in plasma protein profiles associated with inflammation or dysregulated processes were observed following platelet transfusions.

**Discussion:**

Platelet transfusions did not induce specific quantitative changes in plasma protein levels in healthy volunteers. Nonetheless, they were associated with increased levels of PPBP in hemato‐oncological patients. In general, no evidence of inflammation or other dysregulated processes was observed following platelet transfusions.

## 1 INTRODUCTION

Platelet transfusions are commonly administered to treat and prevent bleeding as part of supportive care in trauma,^1^ critically ill,^2^ hemato‐oncological patients^3^ and preterm infants.^4^ Poor correlations between platelet counts and bleeding risks in these patient groups^5^, ^6^ drive an ongoing debate concerning optimal practices regarding dosage,^7^, ^8^, ^9^ transfusion triggers,^6^, ^9^, ^10^, ^11^ and transfusion production methods.^12^, ^13^, ^14^ This debate is fueled by the notion that adverse events linked to platelet transfusions are 3.25 times more frequent when compared to red blood cell transfusions,^15^, ^16^ especially under inflammation and sepsis.^17^, ^18^, ^19^ Moreover, it has been recently shown that inflammatory molecules, including CXCL5, CD40, and TGF‐β are increased upon administration of platelet transfusions in a neonatal population.^20^ These findings align with results from the PlaNet‐2/MATISSE study, which reported significantly higher rates of death or major bleeding in preterm infants treated under a liberal platelet transfusion protocol.^21^ Similar results have also been reported in patients with acute intracerebral hemorrhage undergoing antiplatelet treatment, where platelet transfusions were associated with a higher number of adverse events and odds of death.^22^

Platelet storage lesions, which result from preparation methods, storage duration, and storage medium of platelet concentrates, are associated with an increase of inflammatory‐ and platelet‐derived cytokines as well as platelet activation markers.^23^, ^24^, ^25^, ^26^ This has been suggested to reduce the efficacy of platelet transfusions in patients with inflammation^27^ given the recognized roles of platelets in immunity and inflammation.^28^, ^29^ This highlights the need to investigate the relationship between platelet transfusions and inflammation at the molecular level.

Therefore, this study aimed to investigate transfusion‐induced changes on plasma proteins in adults under inflammatory conditions. To achieve this, we took advantage of controlled endotoxemia as a model of systemic inflammation in which we characterized the plasma proteomic response to LPS. We subsequently evaluated changes on plasma proteomes induced by platelet transfusions both in steady state conditions as well as in conditions of controlled endotoxemia. In addition, we investigated the effect of allogenic platelet transfusions in hemato‐oncological patients,^30^ by comparing post‐ and pre‐transfusion samples.

## 2 METHODS

This study utilizes plasma samples from two previously conducted studies.^31^, ^32^, ^33^

### 2.1 Human healthy controls and human controlled endotoxemia study samples

Plasma samples (*n* = 252 ethylenediaminetetraacetic acid, EDTA) corresponding to the 36 inclusions from the “Development of a ‘two‐hit’ *in vivo* autologous platelet transfusion model in healthy volunteers” (DIVA, NL‐OMON55634) study were included^31^, ^32^ (Table S1, Appendix S1).

### 2.2 Hemato‐oncologic study samples

EDTA plasma samples available from hemato‐oncological patients of an observational cohort study^33^ part of the Pathogen Reduction Evaluation & Predictive Analytical Rating Score (PREPAReS) trial^13^, ^34^ were selected (Table S2, Appendix S1). A total of 14 patients undergoing 27 transfusions (54 samples) were included.

### 2.3 Plasma proteomics workflow

All samples were processed in batches per study. Quality control (QC) samples, pooled plasma samples from 30 healthy donors (Sanquin, the Netherlands), were included in triplicates per 96‐well plate to evaluate inter‐ and intra‐experimental robustness. Plasma samples were thawed at room temperature, and proteins were denatured, alkylated, reduced, and digested into peptides, see Appendix S1. Approximately 500 ng of tryptic digests were analyzed with MS‐based proteomics, operating in data independent mode with parallel accumulation serial fragmentation (diaPASEF, Appendix S1).

### 2.4 Data processing

The data acquired with diaPASEF was processed with DIA‐NN (v1.8.1)^35^ separately for both cohorts using cohort‐specific spectral libraries. For spectral libraries generation, DIA‐NN settings and data processing details see Appendix S1. DIA‐NN matrices (pr‐matrix, pg‐matrix) were loaded into R^36^ and data were log_2_ transformed and imputed using a normal distribution (width = 0.3; downshift = 1.8) (Table S3A–D), which was used for statistical analysis and correlation analysis.

### 2.5 Data analysis

Analytical performance was determined by coefficients of variation (CVs) calculated from non‐log_2_ transformed data of repeated injections from QC samples. In total, 91.1% (healthy volunteer samples) and 89.5% (hemato‐oncological samples) of the proteins were quantified below 30% CV (Figure S1). Principal component analysis (PCA) was performed with prcomp. Statistically different levels of proteins between groups were calculated with moderate *t*‐tests using Limma^37^ with a block on individuals. Protein levels with an absolute log_2_ fold change ≥1 and Benjamini–Hochberg adjusted *p*‐value <.05 were considered significant. Co‐expression analysis of proteins was based on Pearson correlations with the Hmisc package^38^ and protein networks with Pearson coefficients >0.6 were visualized in Cytoscape.^39^ Standardization of data was performed by calculating *Z*‐scores, re‐scaling data with a mean of 0 and standard deviation of 1, to assess changes in protein levels taking the overall mean as a reference. Functional enrichment analysis was performed with the plugin ClueGO version 2.5.10^40^ in Cytoscape (Appendix S1). Inter‐individual variation analysis was performed based on CVs over longitudinal timelines of samples and the average difference of protein levels at all timepoints to baseline for each individual person and protein. A two‐fold difference and 50% CV cut‐off were used to define proteins altered over time. Spearman correlations between protein levels, laboratory, and platelet concentrate parameters were performed with the Hmisc package.

## 3 RESULTS

### 3.1 Platelet transfusion products do not alter plasma protein levels in healthy volunteers

**FIGURE 1:**
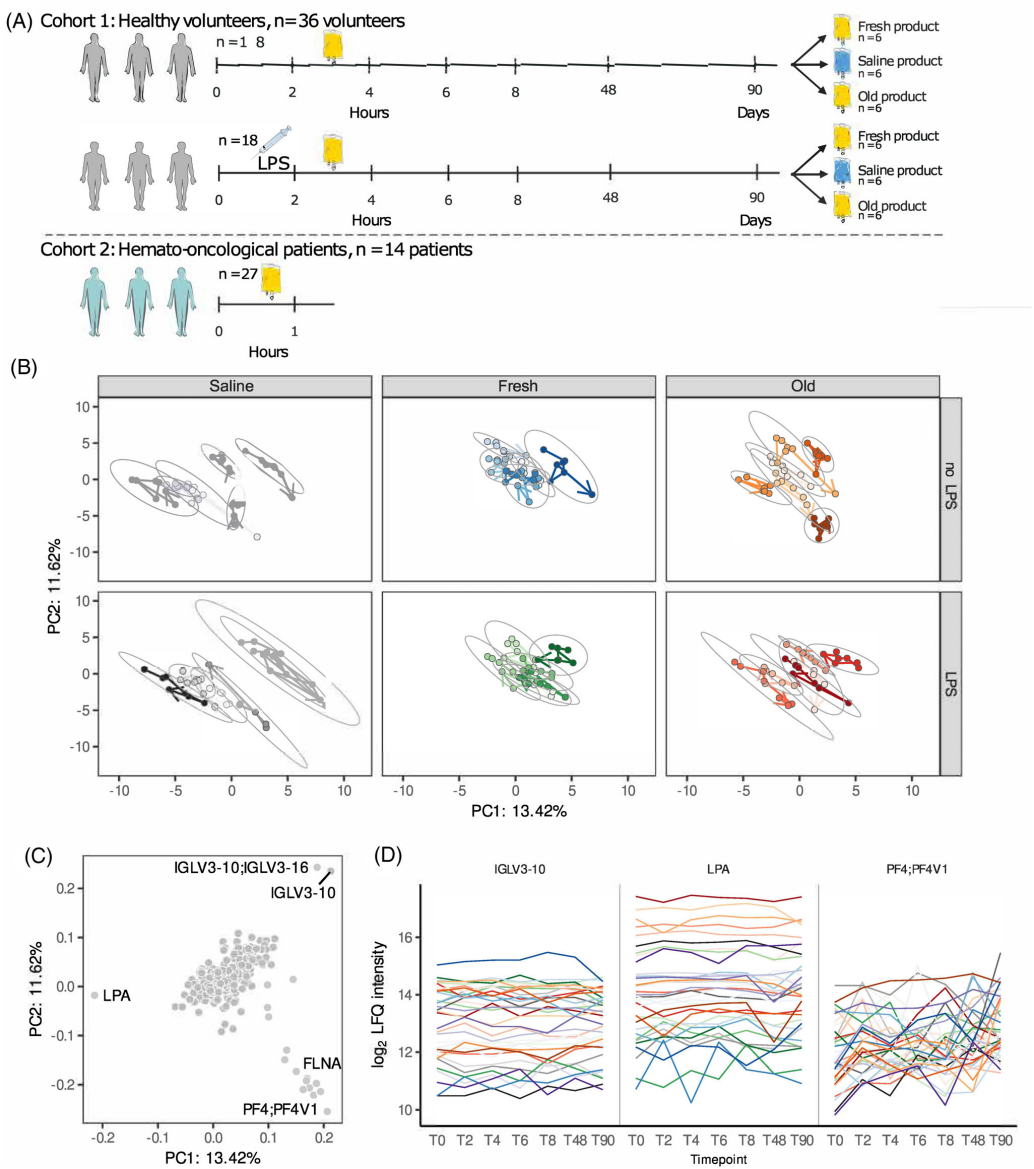
Global plasma proteome profiles reflect inter‐individual heterogeneity. (A) Schematic figure of cohorts studied. (B) Principal component analysis (PCA) results stratified by LPS‐exposure and platelet transfusion product group. Volunteers are depicted by different colors and samples are connected by an arrow depicting their timelines. Ellipses are drawn enclosing all samples from a volunteer. (C) Scatted plot depicting proteins contributions to PC1 and PC2 with the top‐10 proteins with largest contributions labeled by gene name. (D) Log_2_ transformed label free quantification (LFQ) levels of immunoglobulin lambda variable 3–10 (IGLV3‐10), apolipoprotein A (LPA) and platelet factor 4 (PF4;PF4V1) plotted over time for each volunteer, annotated by color. [Color figure can be viewed at wileyonlinelibrary.com]

The impact of autologous platelet transfusions on global plasma proteome profiles of volunteers under healthy or controlled endotoxemia conditions was assessed. This analysis indicated clustering of individuals, regardless of collection timepoint or platelet transfusion group (saline, fresh or old platelets) (Figure 1A). The main proteins contributing to separation along PC1 and PC2 included variable regions of immunoglobulins and apolipoprotein A (LPA) (Figure 1B). The levels of these proteins showed high variability between volunteers, but constant levels throughout time per individual. Since volunteers were included in the study twice (with a year apart), we compared these duplicated inclusions. Levels of LPA and immunoglobulin lambda variable 3–10 (IGLV3‐10) remained constant for each individual independent of time between inclusions and exposure to LPS (Figure S2). In contrast, protein levels of platelet factor 4 (PF4;PF4V1), another main contributor along PC1 and PC2, varied within and between individuals (Figure 1C). These results suggest that inter‐individual variation captured in plasma protein profiles persisted independent of time between study inclusions and study group allocation, while no global trends derived from platelet transfusions in the plasma proteome were observed.

**FIGURE 2:**
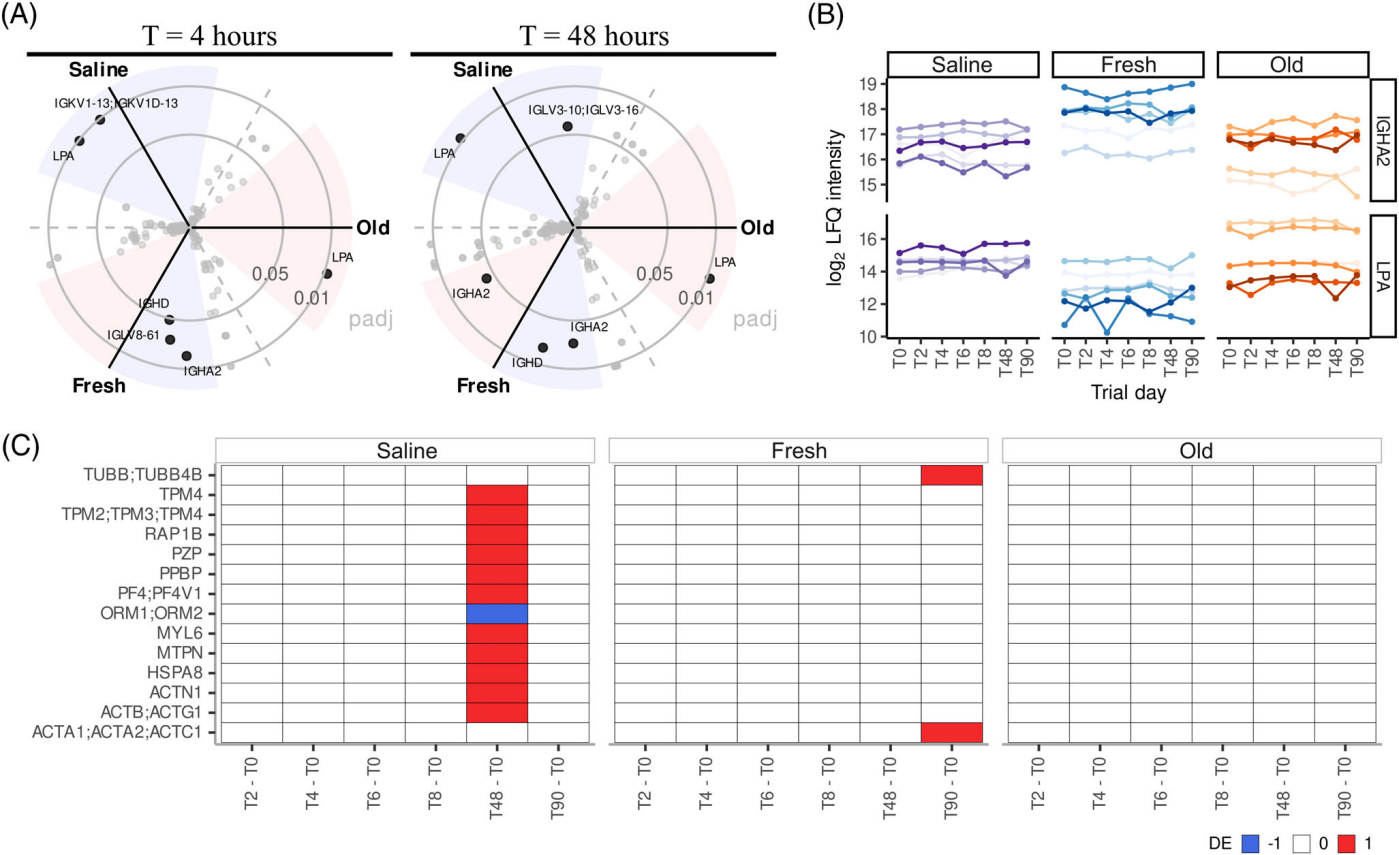
Platelet transfusion do not alter plasma proteome profiles of healthy volunteers. (A) Three‐way radial plots where each side correspond to a volcano plot comparing platelet product groups at T4 and T48 (2 and 48 h after transfusion, respectively). Adjusted p values on y axis and angle for y axis defined based on log_2_ fold‐change (LFC). Shaded areas represent LFC < −1 in blue and >1 in red. (B) Log_2_ transformed label free quantification (LFQ) levels throughout time for example proteins of statistically significant results, lipoprotein A (LPA) and immunoglobulin J chain (JCHAIN). (C) Heatmap representing statistically significant proteins at different timepoints compared to baseline in healthy volunteers with upregulated proteins (red) and downregulated proteins (blue). [Color figure can be viewed at wileyonlinelibrary.com]

The impact of autologous platelet transfusion on plasma proteome profiles of healthy volunteers was evaluated by comparing protein levels between platelet transfusion groups at each timepoint. Nine significant proteins were identified in these comparisons (Table S3E). At 2 h after transfusion (T4) immunoglobulin heavy constant alpha 2 (IGHA2), immunoglobulin lambda variable 8–61 (IGLV8‐61), immunoglobulin heavy constant delta (IGHD) and LPA were significantly different between fresh and old platelet transfusion products, and immunoglobulin kappa variable 1D‐13 (IGKV1‐13; IGKV1D‐13) and LPA were significantly different between fresh platelet transfusions and the saline control group. Comparable results were observed at 48 h after transfusion (T48) (Figure 2A). The levels of IGHA2 and LPA remained constant through time for each individual but varied between volunteers (Figure 2B). Thus, the main differences observed in healthy volunteers after transfusion were driven by inter‐individual variation and not associated with autologous platelet transfusions.

Longitudinal changes of plasma proteins induced by platelet transfusions were investigated by comparing protein levels in samples collected prior to transfusion and LPS (T0) to after transfusion (Table S3F). Few changes in protein levels were captured following transfusions. Moreover, no significant differences were identified in those individuals that received 7‐day stored autologous platelet transfusion products. Most significant alterations were captured at 48 h after transfusion in the control group that received saline. This included proteins such as platelet basic protein (PPBP) and shared regions, peptides shared between proteins, of platelet factor 4, platelet factor 4 variant 1 (PF4; PF4V1) and tropomyosin alpha‐2, ‐3, and ‐4 chain (TPM2; TPM3; TMP4) (Figure 2C). However, these proteins were not significant when comparing all time points against the post LPS and prior to transfusion sample time point (T2) (Figure S3A–C; Table S3F). Therefore, the significant results observed might be due to low numbers in the study groups analyzed. Nonetheless, this suggests that platelet transfusions do not have a major impact on plasma profiles regardless of product storage time.

### 3.2 Controlled endotoxemia triggers a time‐specific and conserved response in plasma protein profiles

**FIGURE 3:**
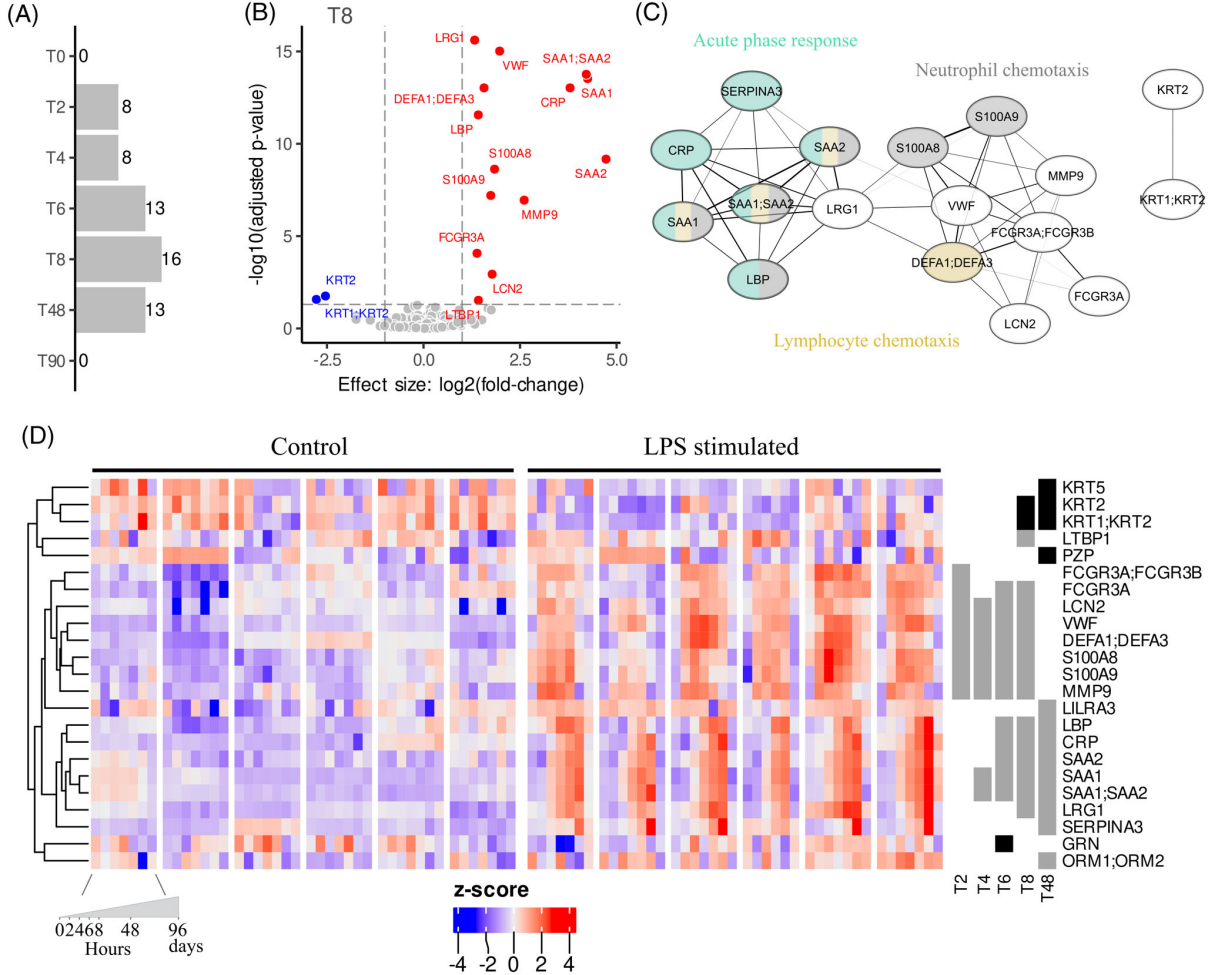
Time‐specific response to LPS in humans under controlled endotoxemia. (A) Barplot with the total number of significant proteins between individuals exposed to LPS compared to those without LPS. (B) Volcano plot for 8 h after LPS exposure (T8) highlighting the significant proteins between individuals exposed to LPS compared to those without LPS. (C) Pearson correlation coefficients network of significant proteins. Annotation of biological pathways are denoted with different colors and white color was assigned to proteins which were not present in pathways based on enrichment thresholds applied. (D) Heatmap depicting standardized intensities, *Z*‐scored, of all significant different proteins in all non‐LPS and LPS exposed volunteers per timepoint. [Color figure can be viewed at wileyonlinelibrary.com]

Previous reports have shown that platelet transfusion efficacy is affected under inflammatory conditions. Therefore, before analyzing changes induced by platelet transfusions under controlled endotoxemia, we first characterized LPS‐induced changes in plasma proteomes in the saline transfusion control group. While no significant differences between these groups were captured either prior to (T0) or 90 days after LPS exposure (T90), significant alterations were observed at 2 (T2), 4 (T4), 6 (T6), 8 (T8) and 48 (T48) hours after LPS exposure (Figure 3A). At all timepoints, most of the significant differences were due to increased protein levels, with the highest number of significantly different proteins detected at T8 (Figure 3B; Table S3G). Clustering based on Pearson correlation coefficients identified two clusters of proteins: cluster A associated with the acute phase, including C‐reactive protein (CRP) and serum amyloid A1 (SAA1), and cluster B associated with neutrophil and lymphocyte chemotaxis, including protein S100A8 (S100A8) and protein S100A9 (S100A9) (Figure 3C). These two clusters were kinetically distinct, with an initial rapid increase in proteins associated with degranulation and the inflammatory response followed by a subsequent rise in acute‐phase proteins (Figure 3D). In summary, controlled endotoxemia in healthy volunteers elicited a shared inflammatory state across individuals, characterized by a time‐dependent response.

### 3.3 Platelet transfusions in humans under controlled endotoxemia induced a limited impact on plasma proteomic profiles

**FIGURE 4:**
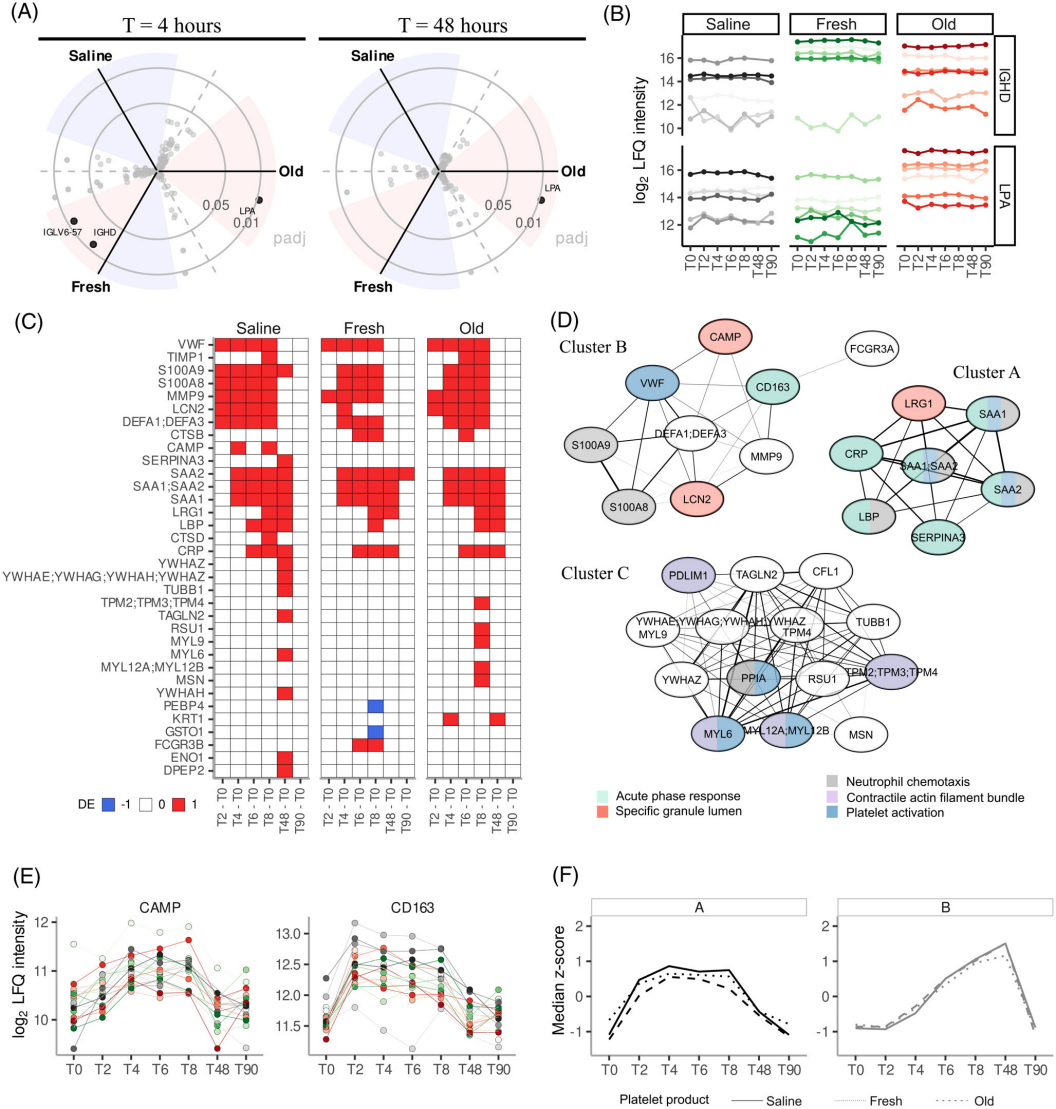
Platelet transfusion does not alter plasma proteome profiles of humans under controlled endotoxemia. (A) Three‐way radial plots where each side correspond to a volcano plot comparing platelet product groups at T4 and T48 (2 and 48 h after transfusion, respectively). Adjusted p values on *y* axis and angle for *y* axis defined based on log_2_ fold‐change (LFC). Shaded areas represent LFC < −1 in blue and >1 in red. (B) Log_2_ transformed label free quantification (LFQ) levels throughout time for example proteins of statistically significant results, lipoprotein A (LPA) and immunoglobulin heavy constant delta (IGHD). (C) Heatmap representing statistically significant proteins at different timepoints compared to baseline in healthy volunteers with upregulated proteins (pink) and downregulated proteins (blue). (D) Pearson correlation coefficients network of significant proteins. Annotation of biological pathways are denoted with different colors and white color was assigned to proteins which were not present in pathways based on enrichment thresholds applied. (E) Log_2_ transformed label free quantification (LFQ) levels for cathelicidin antimicrobial peptide (CAMP) and scavenger receptor cysteine‐rich type 1 protein M130 (CD163) over time for each volunteer under controlled endotoxemia, annotated by color. (F) Median intensities per timepoint calculated from medians of each protein in the cluster. Platelet transfusion group is depicted with different type of line, solid for saline, dotted for fresh and dashed for old. [Color figure can be viewed at wileyonlinelibrary.com]

The impact of autologous platelet transfusion products in an LPS‐induced inflammatory environment was assessed by comparing humans under controlled endotoxemia. Consistent with results in non‐LPS‐exposed volunteers, only immunoglobulin‐associated proteins and LPA were significantly different between transfusion product groups (Figure 4A; Table S3H). These observations were consistent across all time points, suggesting that they are not transfusion product specific (Figure 4B).

Next, longitudinal changes within each platelet transfusion product group were evaluated by comparing protein levels before and after LPS exposure (Figure 4C) and transfusion (Figure S4A; Table S3I). Significant proteins were clustered based on Pearson correlation coefficients (Figure 4D) with similar results as observed in non‐transfused volunteers (Figure 3C). Importantly, two additional proteins were identified in cluster A, cathelicidin antimicrobial peptide (CAMP) and scavenger receptor cysteine‐rich type 1 protein M130 (CD163). Both proteins showed an early upregulation in all patients upon LPS exposure (Figure 4E). Exposure to LPS underpinned similar responses with conserved kinetics independent of platelet transfusion products (Figure 4F). An additional cluster (C) was observed with proteins associated with platelet activation and contractile actin filament bundle (Figure 4D). This cluster had no clear kinetic pattern nor shared changes in protein levels specific to LPS nor transfusions (Figure S4B). This suggested that under inflammatory conditions, platelet transfusions did not induce additional changes in plasma proteome profiles.

**FIGURE 5:**
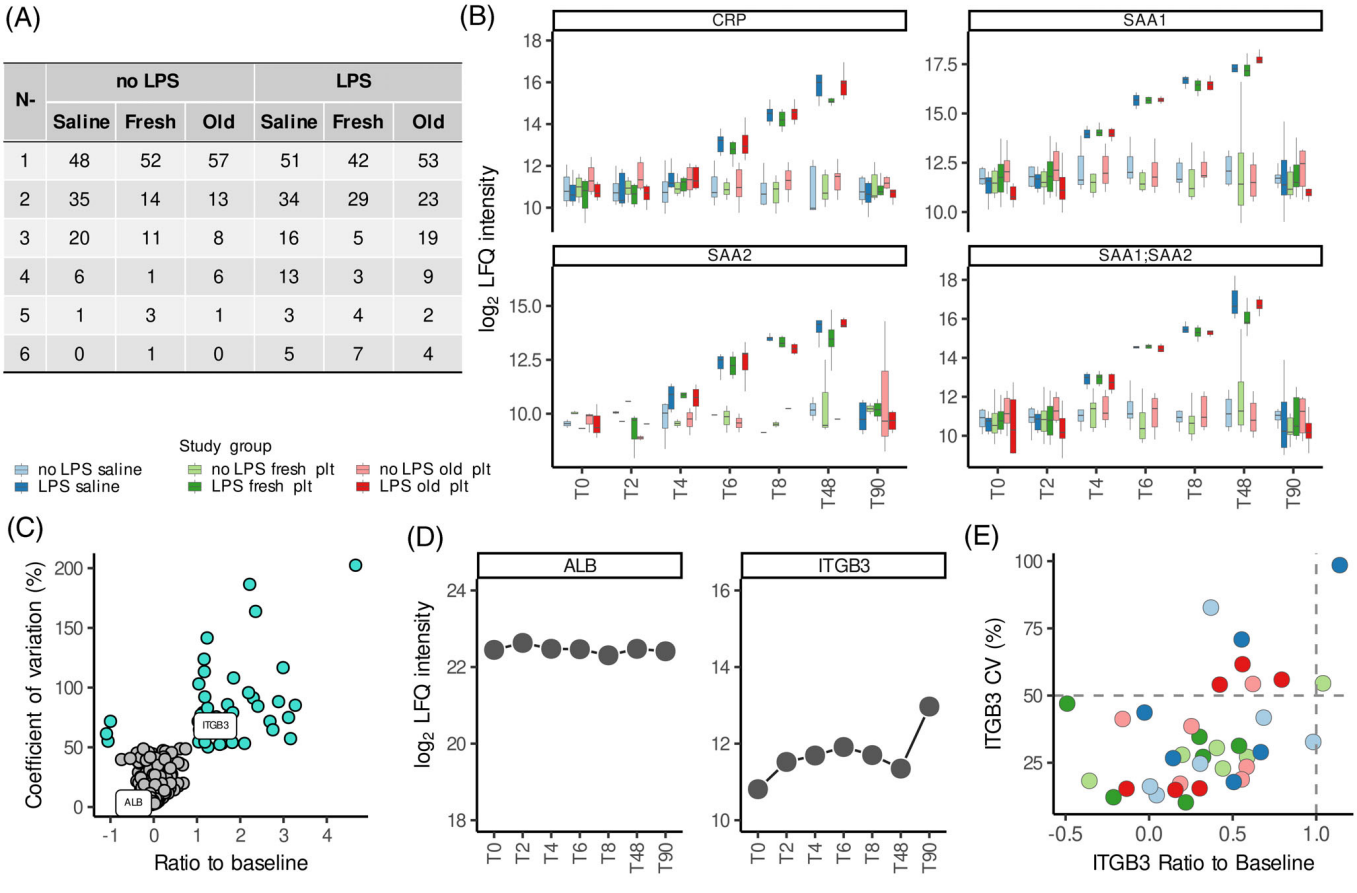
Individual plasma profiles vary through time regardless of transfusion product groups. (A) Table with the number of proteins classified as variable over time (coefficient of variations (CVs) over time >50% and |log_2_ ratio to baseline| >1) summarized by sub‐groups in the study. Number of cases 1–6 represent 1–6 volunteers. (B) Boxplots with the Log_2_ transformed label free quantification (LFQ) levels stratified for transfusion product and LPS exposure of c‐reactive protein (CRP), serum amyloid A1 (SAA1), serum amyloid A2 (SAA2) and peptides shared between SAA1 and SAA2 (SAA1;SAA2), defined as consistently variable in all volunteers under controlled endotoxemia. (C) Scatter plot with CVs over time plotted against the log_2_ ratio of mean LFQ intensities for each protein to their baseline levels for one of the healthy volunteers. Proteins with CVs > 50% and ratio to baseline >1 are highlighted in cyan. (D) LFQ levels throughout time for albumin (ALB) and integrin beta‐3 (ITGB3) for the individual represented in C. (E) Scatted plot with the CVs over time plotted against the log_2_ ratio of mean LFQ intensities for each protein to their baseline levels for ITGB3 per individual. [Color figure can be viewed at wileyonlinelibrary.com]

The persistent influence of individual‐specific protein profiles on our results prompted further analysis utilizing a different, individual‐specific approach. No shared overlap was identified among all healthy volunteers that received transfusions. However, a signature of proteins (CRP, SAA1, SAA1;SAA2 and SAA2) consistently varied across all individuals exposed to LPS (Figure 5A; Table S3J). These proteins increased over time in all controlled endotoxemia groups, regardless of platelet transfusion product (Figure 5B). In contrast, most proteins exhibited individual variability (Figure 5A). For example, in one volunteer, the levels of integrin beta‐3 (ITGB3) had a high CV as it varied over time, whereas albumin (ALB) had a low CV as it remained constant over time (Figure 5C,D). This variation was individual specific, as ITGB3 only showed a high CV and difference to baseline in two out of 36 volunteers (Figure 5E). Taken together, this emphasizes that, in the absence of a strong stimulus, plasma protein profiles tend to be highly individual‐specific over time.

### 3.4 Hemato‐oncological thrombocytopenic patients display individual‐specific plasma proteomic profiles with no strong association to transfusion responsiveness

**FIGURE 6:**
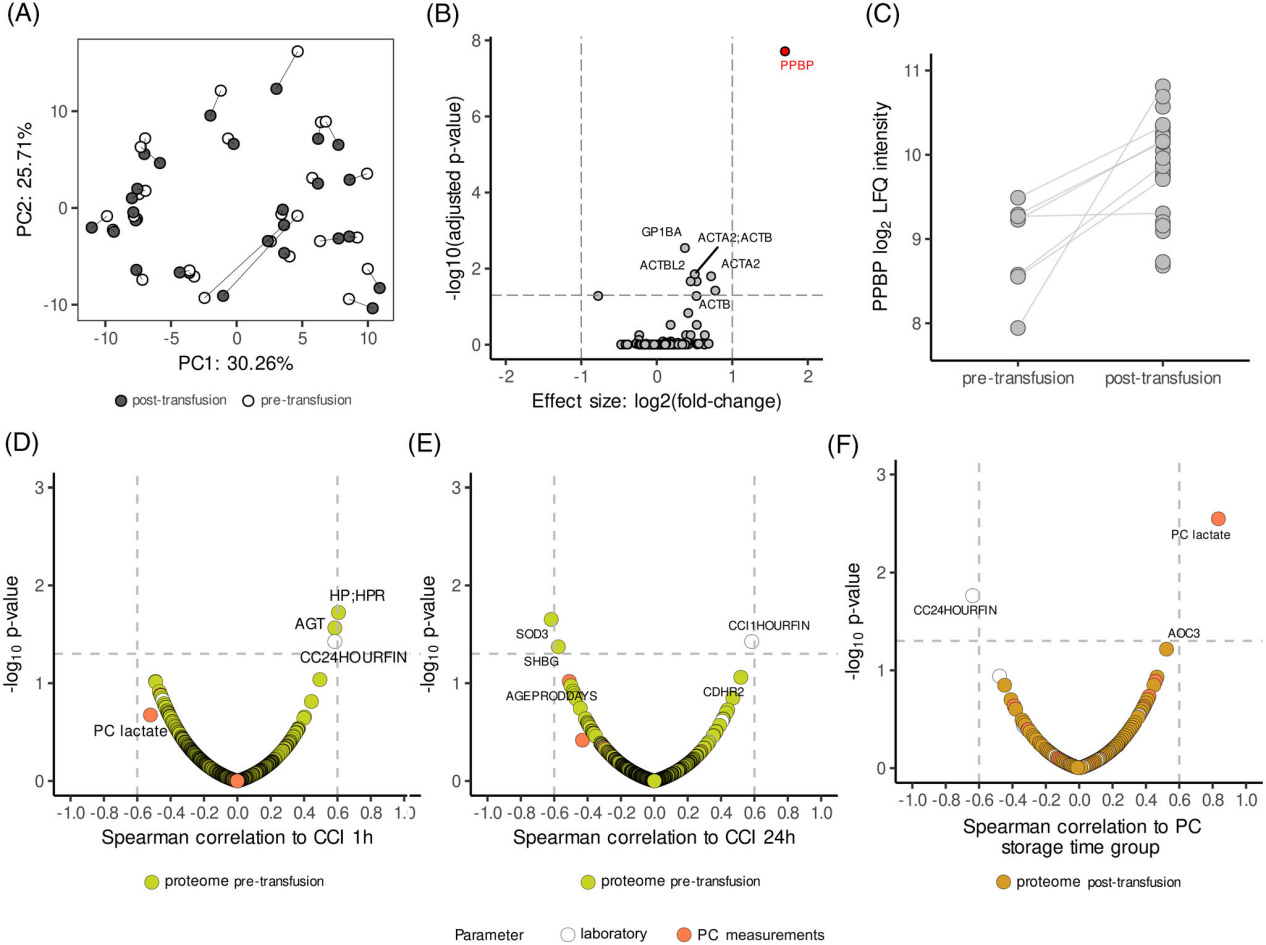
Plasma proteome profiles of hemato‐oncological patients show significant differences post‐transfusion. (A) Principal component analysis (PCA) results with connected paired pre‐ (white) and post‐transfusion samples (black) from hemato‐oncological patients. (B) Volcano plot for statistical comparison of samples pre‐ versus post‐ transfusion highlighting the significant proteins. (C) Log_2_ transformed label free quantification (LFQ) levels for paired pre‐ and post‐transfusion samples connected with lines for platelet basic protein (PPBP). (D) Spearman correlation coefficients between corrected count increment (CCI) 1 h post‐transfusion against and platelet product characteristics measured previously, and all proteins identified pre‐transfusion. (E) Spearman correlation coefficients between corrected count increment (CCI) 24 h post‐transfusion against and platelet product characteristics measured previously, and all proteins identified pre‐transfusion. (F) Spearman correlation coefficients between the storage time of the platelet transfusion product against and platelet product characteristics measured previously, CCIs and all proteins identified post‐transfusion. [Color figure can be viewed at wileyonlinelibrary.com]

To further understand platelet transfusions in a clinical setting, we analyzed samples of hemato‐oncological patients with thrombocytopenia. Plasma proteome profiles before and after platelet transfusions were investigated. PCA again showed clusters by individuals and not storage time (Figure 6A). Protein changes after transfusions revealed a significant increase in PPBP plasma levels post‐transfusion (*p*‐adjust <.05 and |log_2_ fold‐change| > 1). Platelet glycoprotein Ib alpha chain (GP1BA), actin‐aortic smooth muscle (ACTA2) and actin‐cytoplasmic 1 (ACTB) were also significantly different post‐transfusion (p‐adjust <0.05), although they did not meet the effect size threshold (|log_2_ fold‐change| > 1) (Figure 6B; Table S3K). Nonetheless, an increase in protein levels post‐transfusion was observed for all proteins (Figure S4A). Of note, no increase post‐transfusion was observed in the levels of these proteins in samples pre‐ and post‐transfusions from healthy volunteers (Figure S4B).

Next, we sought to investigate whether protein levels associate with platelet transfusion efficacy, using CCI as a proxy. To this end, Spearman correlations between pre‐transfusion plasma levels and platelet product characteristics (i.e., lactate concentration and platelet number) were determined for CCI at 1 h (Figure 6D; Table S3L) and 24 h (Figure 6E; Table S3M) post‐transfusion. CCI at 1 and 24 h were positively correlated to each other (*ρ* = 0.58). Additionally, levels of haptoglobin (HP;HPR, *ρ* = 0.61) and angiotensinogen (AGT, *ρ* = 0.58) positively correlated with CCI at 1 h (Figure 6D), while extracellular superoxide dismutase [Cu‐Zn] (SOD3, *ρ* = −0.62) and sex hormone‐binding globulin (SHBG, *ρ* = −0.57) negatively correlated with CCI at 24 h (Figure 6E).

Also, the influence of platelet product storage time (2–4 and 5–7 storage days) on platelet product characteristics and plasma protein levels post‐transfusion was explored (Figure 6F; Table S3N). Although product storage time was positively correlated with product lactate concentration (*ρ* = 0.83) and negatively correlated with CCI at 24 h (*ρ* = −0.64), no specific associations with plasma proteins in post‐transfusion samples were identified (Figure 6F). Taken together, significant changes in PPBP were observed upon transfusions in hemato‐oncological patients, and CCIs in these patients showed associations with oxidative stress‐related proteins but not with platelet‐associated proteins.

## 4 DISCUSSION

Platelet transfusions are widely used as supportive clinical care to treat or prevent bleeding. Despite efforts to define optimal practices, the molecular effects upon transfusions remain unclear, highlighting the need for thorough evaluation of system‐wide changes induced by platelet transfusions. Therefore, we aimed to understand changes in plasma proteomic profiles upon autologous platelet transfusion in healthy humans, humans under controlled endotoxemia, and in hemato‐oncological patients receiving allogenic platelet transfusions.

Inter‐individual variation was a main contributor to plasma protein levels identified by PCA, emphasized by longitudinal analysis and the identification of significantly different levels of LPA, IGHD, and immunoglobulin associated proteins, all highly individual‐specific.^41^, ^42^ Despite inter‐individual variation, a shared and time‐specific response was observed in humans under controlled endotoxemia. These results are in agreement with a previous report where two main clusters after LPS exposure, (1) an early cluster associated with degranulation detected at 6 h after LPS exposure and (2) a late cluster with mainly liver proteins at 24 h after LPS exposure, were identified.^43^ Importantly, our results highlight previously unreported proteins, including matrix metalloproteinase 9 (MMP9), Fc gamma receptor IIIa and IIIb (FCGR3A and FCGR3A; FCGR3B) and lipocalin‐2 (LCN2), in this early response. MMP9 and LCN2 are neutrophil‐specific proteins important for their anti‐bacterial function,^44^, ^45^ while FCGR3B is critical for neutrophil recruitment under flow conditions^46^ and FCGR3A is an activating molecule of these cells.^47^ Additionally, previous LPS human challenge studies reported an increase of cytokines such as IL‐6, IL‐10, IL‐8, and TNF‐alpha preceding a CRP increase.^48^, ^49^, ^50^, ^51^ These cytokines were not measured in this study, limiting direct comparison with previous findings. However, our results align with the reported early cytokine response linked to leukocyte chemotaxis and activation, highlighting the pivotal early role of these cells in humans under controlled endotoxemia. Importantly, the increase in protein levels captured from both the early (2–8 h after LPS exposure) and late clusters (4–48 h after LPS exposure) were detected earlier than previously reported, 6 and 24 h after LPS exposure, respectively.^43^

The conserved, early, and late, response in controlled endotoxemia was observed in all volunteers, without differences regarding transfusion platelet product. Importantly, no increase of inflammatory cues was observed in healthy volunteers or in hemato‐oncological patients. In addition, we did not observe exacerbation of the inflammatory response in controlled endotoxemia upon transfusion. Taken together, our data showed no evidence of a systemic inflammatory response induced by platelet transfusions, neither in healthy humans nor in humans under controlled endotoxemia or hemato‐oncological patients. This does not align with a previous report where an increase in plasma levels of CXCL5, CD40, and TGF‐β was observed after platelet transfusions in preterm infants,^20^ which could be attributed to the large differences reported between neonates and adults, in immune responses^52^ and hemostasis,^53^ as well as to the differences in proteins identified using Olink inflammation panel and MS‐based approaches.^54^ Future evaluation of cytokines and chemokines in the adult population could help to draw further insights into this topic and these findings should be further verified in larger cohorts and patients with sepsis.

Despite the influence of platelet storage lesion on platelet activation and aggregation, decreased CCI and decreased glucose concentration,^23^, ^24^, ^26^, ^55^ no specific changes in plasma protein levels of healthy volunteers nor hemato‐oncological patients associated with storage time were identified. Yet, in line with previous results, platelet storage time in hemato‐oncological patients had a negative correlation to CCI at 24 h^33^ and a positive association with lactate levels.^56^ A positive association between CCI at 1 h and HP can be explained given the scavenger function of HP, which binds free hemoglobin, preventing platelet activation by hemoglobin itself or by its effect reducing nitric oxide availability.^57^, ^58^, ^59^, ^60^ Similarly, the negative correlation between CCI at 24 h and SOD3 suggests a lack of increase in platelet numbers due to oxidative stress, as SOD3 expression is often induced by anti‐oxidants^61^ which increase in response to oxidative stress.^62^

This study had important limitations to consider. First, our sub‐groups of healthy volunteers consisted of six volunteers per transfusion/controlled endotoxemia condition, resulting in low statistical power and possibly the underestimation of effects on protein levels. However, the longitudinal design of this study with samples prior to and post LPS exposure and transfusion administration enabled individuals to serve as their own control. Specifically, in the case of plasma proteomics, where intra‐individual variation is smaller than inter‐individual variation, longitudinal study designs are advantageous.^63^ Second, the study design did not allow us to distinguish between allogenic and autologous platelet transfusions. This is an important outstanding question, as supportive care for patients typically involves multiple allogenic transfusions associated with the development of anti‐platelet alloantibodies^64^, ^65^ and the development of adverse events.^66^ However, healthy volunteers cannot be safely exposed to allogenic blood transfusions due to the risk of allosensitization that poses a potential future risk if they require blood transfusions or organ transplantation.^67^ Third, differences in keratin and platelet proteins can be attributed to sample contamination and handling, respectively, which should be considered in the analysis and interpretation of the data. Fourth, healthy volunteers under controlled endotoxemia experienced a mild and temporary thrombocytopenia, which limits the generalizability of the findings to patients with severe thrombocytopenia. Thereby, the low ratio of transfused platelets to native platelets in the study population may have diluted the changes in platelet‐derived protein levels post‐transfusion. This might explain why changes in platelet associated proteins were only observed in hemato‐oncological patients with severe thrombocytopenia (<50 × 10^9^ platelets/L) and not in healthy volunteers in the absence and presence of controlled endotoxemia. Fifth, while LPS‐induced endotoxemia models are useful for studying specific biological mechanisms such as immune responses to LPS and TLR4, LPS alone lacks the complexity of human sepsis. The stimulus in this model is not long lasting as in the case of critically ill patients; thus, it does not fully resemble the pathogenesis of sepsis.^68^ Hence, the effects of transfusions under controlled endotoxemia observed here cannot necessarily be extrapolated to patients' populations undergoing inflammation, such as critically ill patients or neonates, as underlying systemic changes unique to each of these populations could affect plasma protein levels. Lastly, the hemato‐oncological cases analyzed here had different diseases and treatments, and the platelet transfusions studied were administered at different stages of treatment. These different underlying phases of platelet deficiency and overall patient state could have affected our analysis.

Nonetheless, this study is the first to apply plasma proteomics on longitudinally collected samples to dissect the changes induced by platelet transfusions and controlled endotoxemia to further our understanding of their potential synergistic effects. In summary, humans under controlled endotoxemia showed a shared response characterized by the successive increase in plasma levels of two distinct protein clusters. Autologous platelet transfusions with different product storage times did not induce a plasma protein signature in LPS‐exposed and non‐LPS‐exposed healthy volunteers. However, an increase of PPBP was identified after allogenic transfusions in hemato‐oncological patients. Platelet transfusions under inflammatory conditions, controlled endotoxemia, and hemato‐oncological treatment, did not exacerbate the increment of inflammatory protein levels measured in this study.

## AUTHOR CONTRIBUTIONS

E. R. S. and D. M. S. performed proteomics analysis, data analysis, designed figures, and wrote the manuscript. S. F. v. W. recruited subjects from the DIVA, I. V., S. F. v. W., and F. L. F. v. B. provided study material from the DIVA. A. L. P. and A. P. J. V. were responsible for the study conception and design of the DIVA study; P. v. d. M., P. F. Y., and J. L. H. K. handled study conception and design, clinical data collection, and provided study material from the PATS. J. V. supervised the research. M. v. d. B. and D. M. S. designed the study and supervised the research. E. R. S., D. M. S., and M. v. d. B. interpreted the data. I. V., J. V., and M. v. d. B. critically reviewed the manuscript. All authors critically read the manuscript and approved the final manuscript.

## FUNDING INFORMATION

The current study was funded by Sanquin PPOC Grant 22_03. The DIVA study is funded by ZonMW (Zorgonderzoek Medische Wetenschappen), part of the NWO (Nederlandse Organisatie voor Wetenschappelijk Onderzoek; the Dutch Organization for Scientific Research, Den Haag, the Netherlands), project number 843002625 and project 09150172010047 and Sanquin PPO 18‐06. The PATS study was funded by Sanquin PPOC Grant 11‐003.

## CONFLICT OF INTEREST STATEMENT

The authors have disclosed no conflicts of interest.

## ETHICS STATEMENT

The DIVA study was approved by the medical ethical committee of the Amsterdam UMC (reference number 2014_294#B2014961). All volunteers gave their written informed consent according to the Declaration of Helsinki. The DIVA study is registered at the World Health Organization International Clinical Trials Registry Platform (NL‐OMON55634). The PATS study was approved by the medical ethical committees of Haga Teaching Hospital (The Hague, the Netherlands), Leiden University Medical Center (Leiden, the Netherlands) and by the Scientific Committee of the Center for Clinical Transfusion Research, Sanquin Leiden, the Netherlands. The PATS study was an observational cohort study part of the PREPAReS trial, which was sponsored by Sanquin Blood Supply and registered at the Netherlands National Trial Registry under number NTR2106, as well as at clinicaltrials.gov under number NCT02783313.

## CONSENT

Informed consent was obtained from patient(s).

## Supporting information


**APPENDIX S1.** Supporting information.

**TABLE S3.** Supporting information.

## Data Availability

The datasets used and/or analyzed during the current study are available in the ProteomeXchange/PRIDE archive database with identifier PXD060882.
