# Supplementary material for: Impact of platelet transfusions on plasma proteomes in controlled endotoxemia and in hemato‐oncological patients
Source: Transfusion. 2025 Sep 3;65(10):1825–38. doi: 10.1111/trf.18397 (PMC12531923; doi:10.1111/trf.18397)
Supplement: Supplementary file 1 — APPENDIX S1. Supporting information. [file TRF-65-1825-s002.docx]

**Impact of Platelet Transfusions on Plasma Proteomes in Controlled Endotoxemia and in Hemato-Oncological Patients**

Eva R. Smit^1^, Isabella Viegen^2^, Pieter F. van der Meer^3,4^, Stefan F. van Wonderen^2,5^, Floor L.F. van Baarle^2,5^, Paula F. Ypma^3^, Jean-Louis Kerkhoffs^3,6^, Jan Voorberg^1,7^, Alexander P.J. Vlaar^2,5^, Anna L. Peters^2^, Maartje van den Biggelaar^1^, Diana Muñoz Sandoval^1^

This file contains supplementary methods, tables and figures.

Table of Contents

[Supplementary methods 2](#_Toc195186552)

[Human healthy controls and human controlled endotoxemia study samples 2](#_Toc195186553)

[Haemato-oncologic study samples 2](#_Toc195186554)

[Plasma sample preparation 3](#_Toc195186555)

[Plasma proteomic LC-MSMS analysis 4](#_Toc195186556)

[Spectral library generation 3](#_Toc195186557)

[Data processing 4](#_Toc195186558)

[Data analysis 5](#_Toc195186559)

[Supplementary Tables 6](#_Toc195186560)

[Supplementary Table 1 6](#_Toc195186561)

[Supplementary Table 2 7](#_Toc195186562)

[Supplementary Figures 8](#_Toc195186563)

[Supplementary Figure 1 8](#_Toc195186564)

[Supplementary Figure 2 9](#_Toc195186565)

[Supplementary Figure 3 10](#_Toc195186566)

[Supplementary Figure 4 11](#_Toc195186567)

[Supplementary Figure 5 12](#_Toc195186568)

# Supplementary methods

## Human healthy controls and human controlled endotoxemia study samples

The DIVA study included healthy volunteers who received either LPS (n=18) or saline (n=18). Subsequently, they received an autologous transfusion with either platelets short-term stored for 2 days (fresh, n=6), long-term stored for 7 days (old, n=6) or saline (n=6). Blood samples were collected at seven different time points: before and after LPS administration as well as before and after transfusion. EDTA plasma collection was described previously(1,2), and samples were stored at -80 °C until further analysis. This study included single participations (n=12) and duplicated participations (n=12, collected 1-2 years apart). This study was approved by the medical ethical committee of the Amsterdam UMC (reference number 2014_294#B2014961). All volunteers gave their written informed consent according to the Declaration of Helsinki. The study is registered at the World Health Organization International Clinical Trials Registry Platform (NL-OMON55634).

## Haemato-oncologic study samples

The observational cohort study within the PREAPReS trial was approved by the medical ethical committees of Haga Teaching Hospital (The Hague, the Netherlands), Leiden University Medical Center (Leiden, the Netherlands) and by the Scientific Committee of the Center for Clinical Transfusion Research, Sanquin Leiden, the Netherlands. EDTA plasma collection was described previously(32), and samples were stored at -80 °C. Patients that received standard platelet concentrates and had samples collected pre- and post- transfusion available were included. These patients presented different diseases, namely acute myeloid leukaemia, acute lymphoblastic leukaemia, chronic myeloid leukemia, multiple myeloma, Hodgkin’s lymphoma and non-Hodgkin’s lymphoma and received different treatments including remission induction chemotherapy, consolidation chemotherapy, allogeneic stem cell transplant or autologous stem cell transplant. The platelet transfusions analyzed in these patients were administered at different times during treatment, ranging from before up to 27 days after treatment. Information on corrected count increments (CCI) for each transfusion was included when available(33). Platelet concentrate information such as lactate concentration and product age was also included.

## Plasma sample preparation

Frozen plasma samples were thawed at room temperature after which 10 μL of each sample was diluted 1:60 in 100 mM Tris(hydroxymethyl)aminomethane hydrochloride (Tris, Life Technologies, UK) (pH = 8.0). Next, for denaturation, reduction and alkylation 5 μL of 20 mM Tris(2-carboxyethyl)phosphine (Thermo Fisher Scientific, USA) and 80 mM chloroacetamide (Sigma Aldrich, Germany) in 100 mM Tris (pH = 8.0) was added to 9 μL of diluted plasma and the protein mixture was resuspended, heated at 95 °C for 5 min and cooled down to RT. Protein digestion was performed with 200 ng mass spectrometry (MS)-grade trypsin Gold (Promega, Madison, WI) in 50 mM tris(hydroxymethyl)aminomethane hydrochloride (Tris, life technologies, UK). After digestion, peptides were acidified with 5 μL of trifluoroacetic acid (TFA, Thermo Fisher Scientific, USA) to a final concentration of 1% (v/v) and samples were frozen at -20 °C until analysis.

## Spectral library generation

Study-specific spectral libraries were generated by pooling aliquots of peptide mixtures of all included study samples, of which 2.5 mL of pooled aliquots were fractionated using in-house prepared StageTips with 3 layers of EmporeTM styrene divinyl benzene-reverse phase sulfonate (SDB-RPS, Supelco, Bellefonte, PA) adapted from Kulak et al.(3). First, stageTips were washed with 65 µL acetonitrile (ACN, BioSolve, the Netherlands) and with 65 µL 0.2% TFA. Next, samples were loaded onto stageTips, twice washed with 65 µL 0.2% TFA and fractionated with 50 µL of (1) 75 mM ammonium formate (AF, Thermo Fisher Scientific, Rockford, IL) in 30% ACN, (2) 90 mM AF in 35% ACN, (3) 100 mM AF and 0.5% FA in 40% ACN, (4) 117 mM AF and 0.5% FA in 45% ACN, (5) 133 mM AF and 0.5% FA in 55% ACN, (6) 150 mM AF and 0.5% FA in 60% ACN, (7) 160 mM AF and 0.5% FA in 68% ACN and (8) 2x 30 µL of 5% ammonium hydroxide (Merck, Germany) in 80% ACN. In between each fraction samples were centrifuged at 200g for until all buffer had eluted, after which the peptides were dried at 30 °C under vacuum in a speedvac and then reconstituted in 0.1% FA in water (BioSolve, the Netherlands).

Samples were loaded onto Evotip PureTips (Evosep, Denmark) according to manufactures guidelines and analyzed using an Evosep One liquid chromatography (LC) system (Evosep, Denmark)(4) using the pre-defined extended 15SPD on a 15 cm column (EV1137, Evosep, Denmark) with ion mobility fractionation gradient on a timsTOF HT (Bruker Daltonics, Billerica, MA) equipped with a CaptiveSpray ionization source at 1600 V. Solvent A was 0.1% formic acid in water and solvent B 0.1% formic acid in acetonitrile.

Spectral library data was acquired in data dependent acquisition with Parallel Accumulation Serial Fragmentation (ddaPASEF) mode. This method had a 1.17 second cycle time, precursors were selected based on the default ion cloud filter with 10 PASEF ramps. The following settings were used: accumulation time set to 100 ms, duty cycle to 100%, target intensity to 20000, with a threshold of 2500 and collision energy as a linear function of 1/k0. For Ion Mobility Fragmentation, the mobility ranges were set as follows: 0.6-0.8, 0.8-1.05,0.9-1.15, 1-1.25, 1.2-1.45,1.35-1.6 1/k0.

For both cohorts a spectral library was made in FragPipe (v20.0)(5–7) using MSFragger (v3.8), IonQuant (v1.9.8)(8) and Philosopher (v5.0.0)(9) using default settings. For the FASTA sequence database, the reviewed human proteome database (Swiss-Prot Database, 20423 entries, downloaded on 8 August 2023) was used, including decoys (reverse) and common contaminants.

## Plasma proteomic LC-MSMS analysis

Approximately 500 ng of tryptic digests from the plasma samples of both studies were loaded onto EvoTip PureTips simultaneously using manufacturer’s guidelines and stored at 4°C until liquid chromatography MSMS analysis. Samples were analyzed using the same instrumental setup as used for the spectral library on a 8 cm column (EV1109, Evosep, Denmark) using the pre-defined 60 SPD method with the same solvents as used for the spectral library. The MS operated in data independent acquisition with Parallel Accumulation Serial Fragmentation (diaPASEF) with an in-house pydiAID(10) optimized method on a timsTOF HT. This method consisted of 16 isolation windows from 302.68 m/z to 1449.72 m/z and 1/K0 0.70 to 1.45 V*s cm−2. The cycle times was 1.85 seconds with collision energy, ramp time and duty cycle as stated for the spectral library.

## Data processing

For quantification, data was processed with DIA-NN (v1.8.1)(11) default settings, following the robust LC (high precision) quantification strategy with a set mass accuracy of 15 ppm, a peptide length between 6-30 amino acids, match between runs enabled, heuristic protein inference disabled, up to two missed cleavages and three variable modifications. Reannotate was enabled based on the same reviewed human proteome database for the spectral library.

Label free quantification (LFQ) intensities are the relative amounts of proteins identified between samples. These values are used to compare protein quantities between samples. Only LFQ values based on 2 or more unique precursors per protein group were kept. Proteins present in at least 50% of all samples within each study, stratified by LPS exposure and platelet product, in the healthy volunteers and all samples from the haemato-oncological patients were considered as quantified.

## Data analysis

Proteins were annotated using gene ontology terms from biological processes, cellular component, and molecular function. The parameters used to run ClueGo were: GO tree interval 3-8, minimum number of genes per cluster 3 as a minimum of 4% from a pathway. Only pathways with a p value < 0.05 were kept. Statistical and grouping parameters were set as default. Biological pathways illustrated in each cluster were defined as the top term with >2 number of genes, GOlevel >5, and lowest term corrected p-value in each GOGroup.

**Supplementary Tables**

Supplementary Table 1**: Demographic characteristics from healthy volunteers from the DIVA study**

Supplementary Table 2**: Demographic characteristics from patients in the PATS study**

# Supplementary Figures


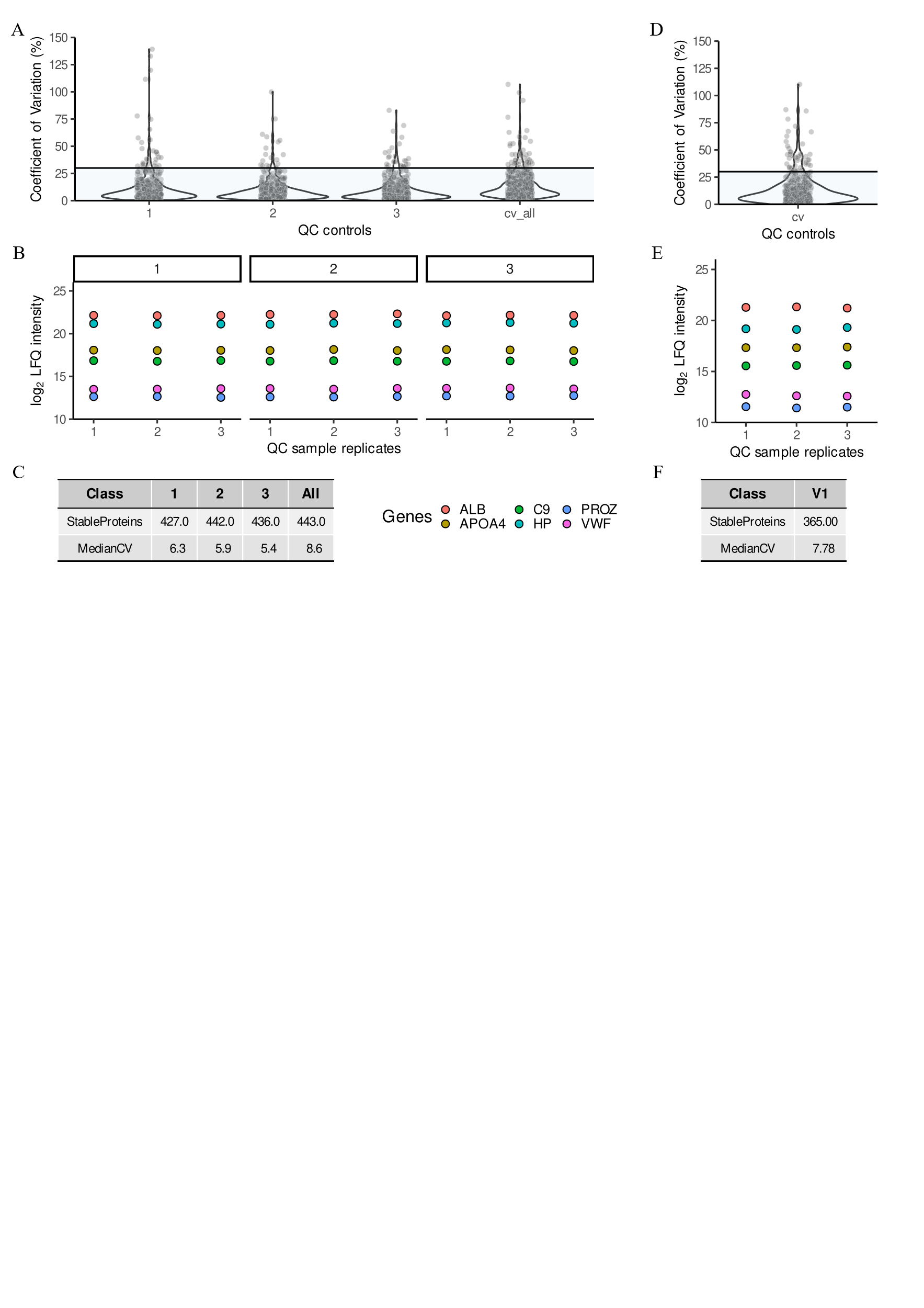


Supplementary Figure 1**. Quality control checks of system and workflow.**

Plasma control samples consisting of aliquots from plasma pool of 30 healthy controls (Sanquin, the Netherlands) were processed along the studies’ samples and used as quality control (QC) samples. The LC-MS system was injected 3 times with QC samples in each plate containing samples. Coefficient of Variation of all QCs from healthy control study **A)** and hemato-oncological samples **D)**. Stability of example proteins in QCs from healthy control study **B)** and hemato-oncological samples **E)**. QC summary results for all plates processed from healthy control study **C)** and hemato-oncological samples **F)**.


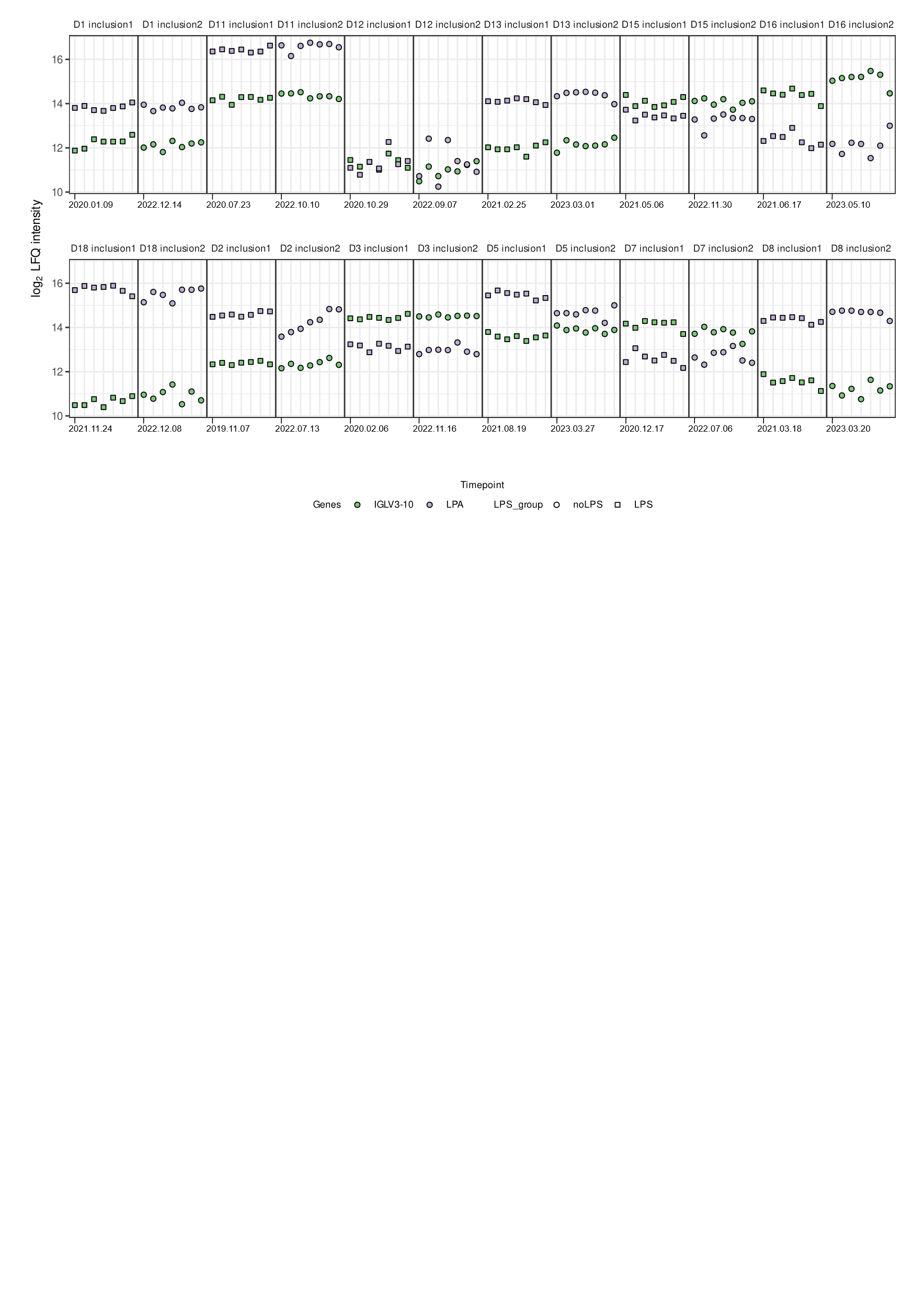


Supplementary Figure 2**. Protein stability in healthy donors across two inclusions.**

Log_2_ label free quantification (LFQ) levels for apolipoprotein-a (LPA, purple) and immunoglobulin lambda variable 3-10 (IGLV3-10, green) from healthy volunteers with two inclusions are shown side by side. Each two panels represent the two inclusions of a volunteer with their start date on the x axis. Vertical lines across the x axis refer to the timepoints in this study, T0, T2, T4, T6, T8, T48 and T90.


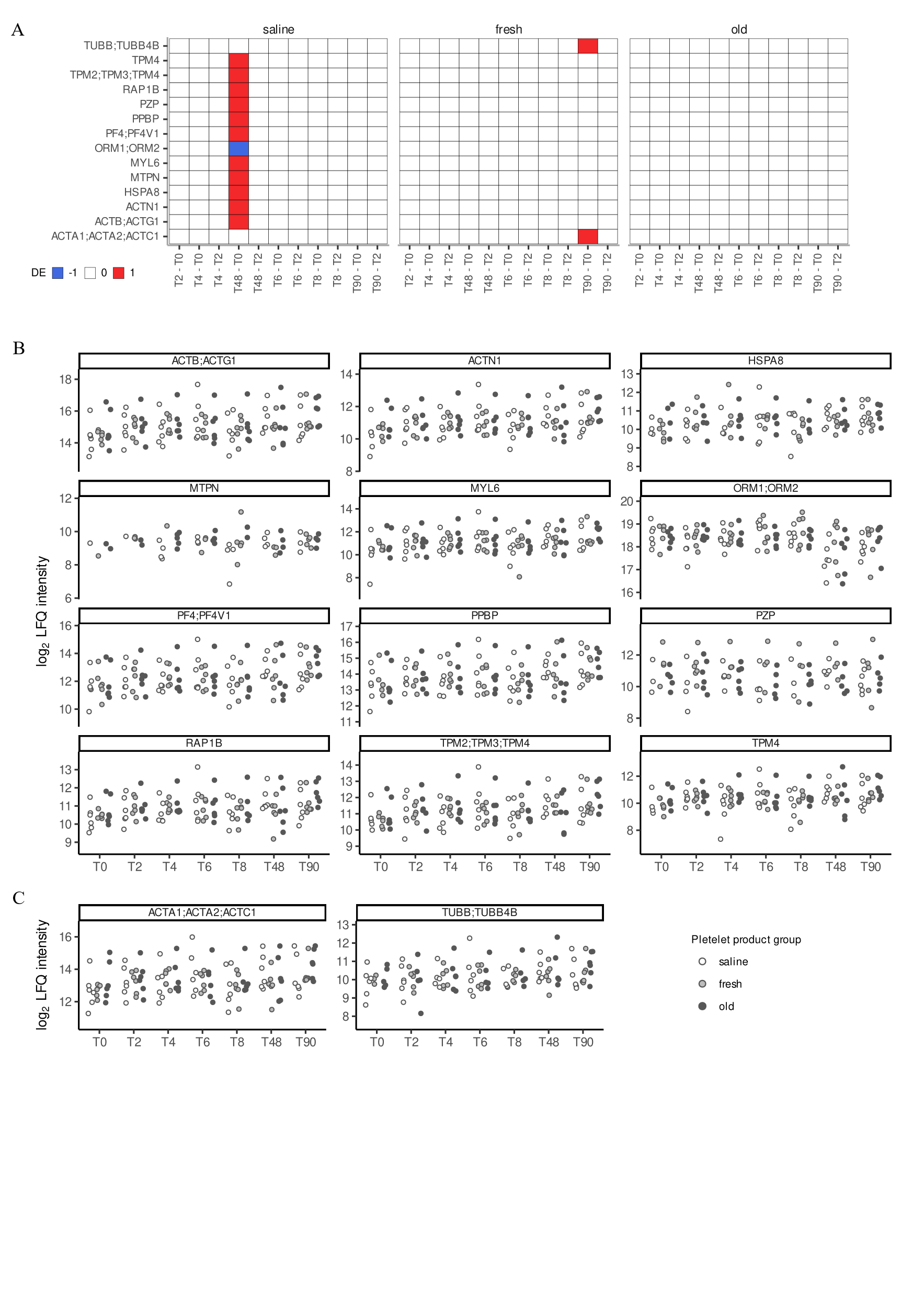


Supplementary Figure 3**. Statistical significant results in transfusion product groups of non-LPS exposed healthy volunteers.**

Log_2_ label free quantification (LFQ) levels of proteins at all timepoints were compared to baseline samples, T0 and T2, for all healthy volunteers without LPS exposure. **A)** Significant results per platelet transfusion group. Up-regulation is depicted in red and down-regulation in blue. LFQ levels of all significant proteins detected in the saline **B)** and old **C)** platelet transfusion group in all volunteers studied. **B-C)** Data is coloured based on platelet transfusion group, white, light grey and dark grey for saline, fresh and old group, respectively.


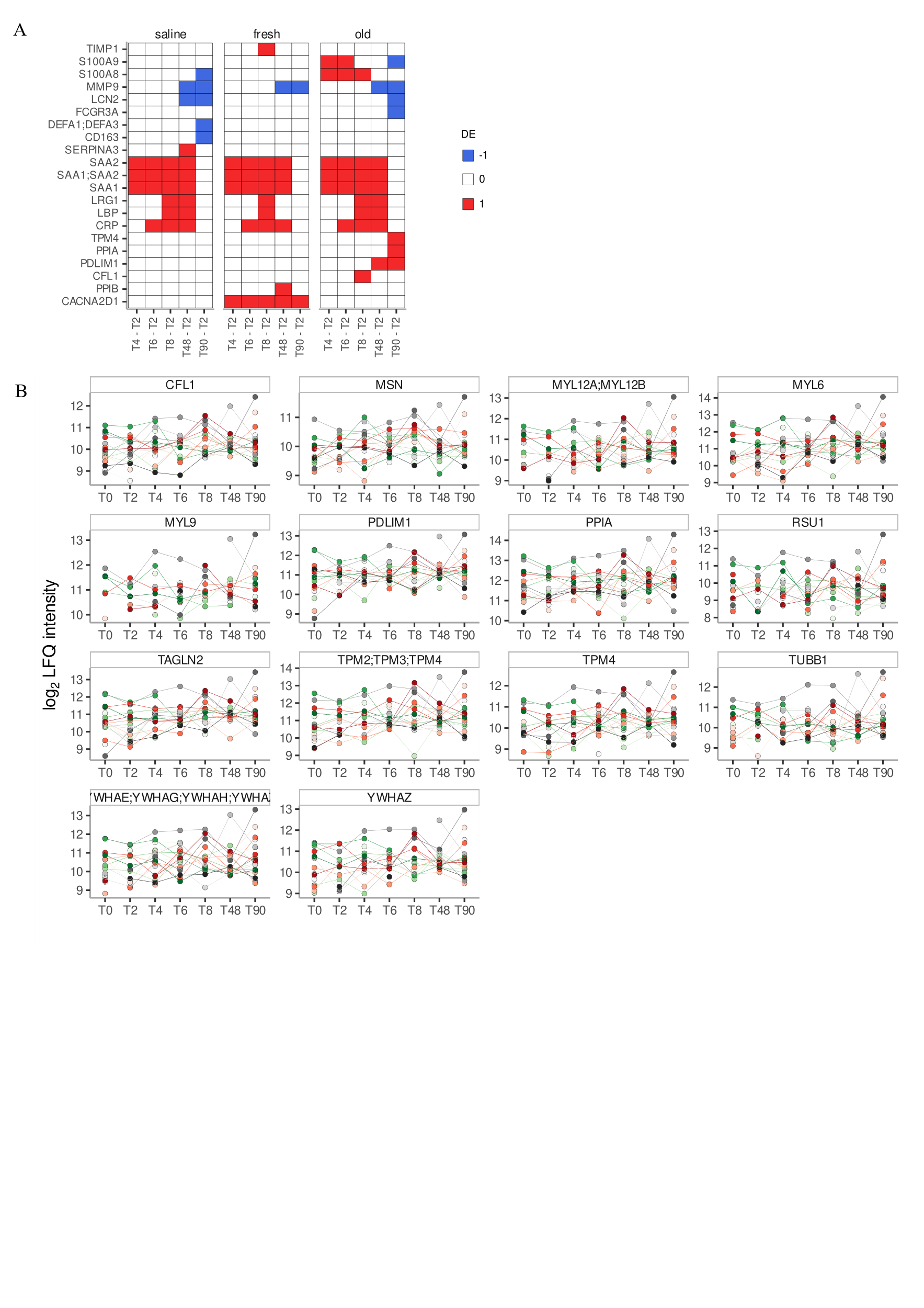


Supplementary Figure 4**. Statistical significant results in transfusion product groups of LPS exposed healthy volunteers.**

Log_2_ label free quantification (LFQ) levels of proteins at all timepoints were compared to baseline samples, T0 and T2, for all healthy volunteers exposed to LPS. **A)** Significant results per platelet transfusion group. Up-regulation is depicted in red and down-regulation in blue. **B)** LFQ levels of significant proteins in cluster C are shown for all volunteers exposed to LPS. Data is coloured based on platelet transfusion group, shades of grey, green and red represent saline, fresh and old group, respectively.


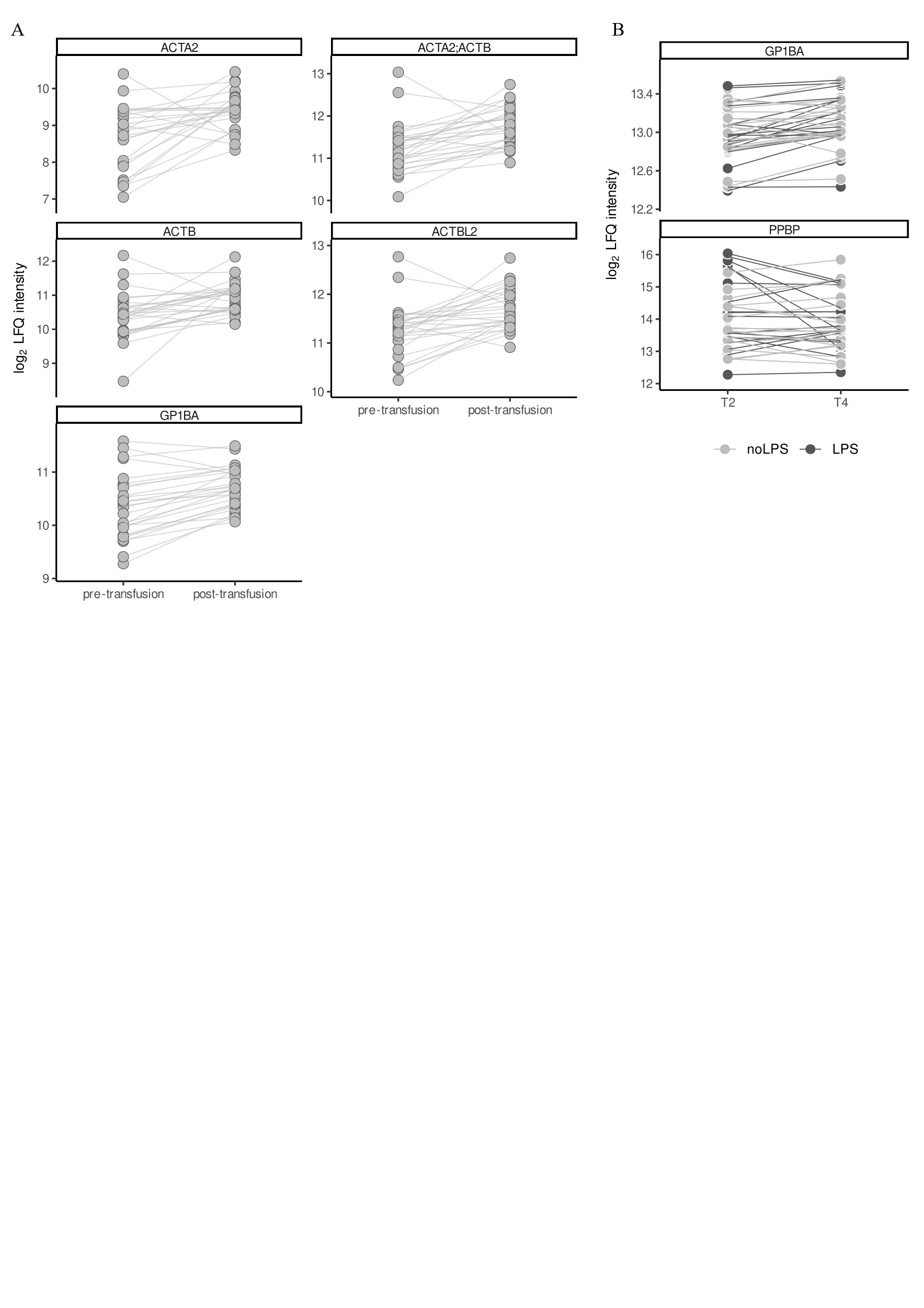


Supplementary Figure 5**. Platelet associated proteins in pre- and post-transfusion samples.**

Platelet associated proteins identified as significantly different but did not meet the effect size criteria of analysis in hemato-oncological patients. Log_2_ label free quantification (LFQ) levels of these proteins in pre- and post-transfusion samples from hemato-oncological patients **A)** and healthy controls **B).** Pre- and post-transfusion samples of each individual are connected with a line. T2 = immediately before transfusion and T4= 2 hours after transfusion. In **B)** groups of healthy volunteers with and without exposure to LPS are shown in dark and light grey, respectively.

References

1. van Baarle FLF, de Bruin S, Bulle EB, van Mourik N, Lim EHT, Tuip-de Boer AM, et al. Aged versus fresh autologous platelet transfusion in a two-hit healthy volunteer model of transfusion-related acute lung injury. Transfusion. 2022 Dec;62(12):2490–501.

2. van Wonderen SF, van Baarle FLF, Tuip-de Boer AM, Polet CA, van Bruggen R, Vermeulen C, et al. Hemostatic conditions following autologous transfusion of fresh vs stored platelets in experimental endotoxemia: an open-label randomized controlled trial with healthy volunteers. Res Pract Thromb Haemost. 2024 Nov;8(8):102612.

3. Kulak NA, Pichler G, Paron I, Nagaraj N, Mann M. Minimal, encapsulated proteomic-sample processing applied to copy-number estimation in eukaryotic cells. Nat Methods. 2014;11(3):319–24.

4. Bache N, Geyer PE, Bekker-Jensen DB, Hoerning O, Falkenby L, Treit P V, et al. A Novel LC System Embeds Analytes in Pre-formed Gradients for Rapid, Ultra-robust Proteomics. Mol Cell Proteomics. 2018 Nov;17(11):2284–96.

5. Teo GC, Polasky DA, Yu F, Nesvizhskii AI. Fast Deisotoping Algorithm and Its Implementation in the MSFragger Search Engine. J Proteome Res. 2021 Jan;20(1):498–505.

6. Kong AT, Leprevost F V, Avtonomov DM, Mellacheruvu D, Nesvizhskii AI. MSFragger: ultrafast and comprehensive peptide identification in mass spectrometry–based proteomics. Nat Methods [Internet]. 2017;14(5):513–20. Available from: https://doi.org/10.1038/nmeth.4256

7. Yu F, Haynes SE, Teo GC, Avtonomov DM, Polasky DA, Nesvizhskii AI. Fast Quantitative Analysis of timsTOF PASEF Data with MSFragger and IonQuant. Mol Cell Proteomics. 2020 Sep;19(9):1575–85.

8. Yu F, Haynes SE, Nesvizhskii AI. IonQuant Enables Accurate and Sensitive Label-Free Quantification With FDR-Controlled Match-Between-Runs. Mol Cell Proteomics. 2021;20:100077.

9. da Veiga Leprevost F, Haynes SE, Avtonomov DM, Chang H-Y, Shanmugam AK, Mellacheruvu D, et al. Philosopher: a versatile toolkit for shotgun proteomics data analysis. Nat Methods [Internet]. 2020;17(9):869–70. Available from: https://doi.org/10.1038/s41592-020-0912-y

10. Skowronek P, Thielert M, Voytik E, Tanzer MC, Hansen FM, Willems S, et al. Rapid and In-Depth Coverage of the (Phospho-)Proteome With Deep Libraries and Optimal Window Design for dia-PASEF. Mol Cell Proteomics. 2022 Sep;21(9):100279.

11. Demichev V, Messner CB, Vernardis SI, Lilley KS, Ralser M. DIA-NN: neural networks and interference correction enable deep proteome coverage in high throughput. Nat Methods. 2020;17(1):41–4.
